# ﻿Exploration of *Hiptage* (Malpighiaceae) diversity in Vietnam reveals a new species with wingless fruits

**DOI:** 10.3897/phytokeys.256.148573

**Published:** 2025-06-02

**Authors:** Mai Thi Xuan Lam, Marie-Stella Jagou, Giang Quoc Van, Florent Martos, Truc Thi Ngoc Nguyen, Truong Van Do, Rafael Felipe de Almeida, Hoa Dang Tran, Khoa Dang Tran, Giang Thi Nguyen, Dong Phuong Tran, Ky Huynh, Jean-Noël Rivière, Mathieu Rouget, Diep Quang Dinh, Anh Tuan Le, Xuan Thi Trinh, Jean-Philippe Deguine, Pierre Lefeuvre

**Affiliations:** 1 College of Agriculture, Can Tho University, Can Tho, Vietnam Can Tho University Can Tho Vietnam; 2 Université de La Réunion, UMR PVBMT, F-97410 St Pierre, La Réunion, France CIRAD, UMR PVBMT La Réunion France; 3 CIRAD, UMR PVBMT, F-97410 St Pierre, La Réunion, France Université de La Réunion La Réunion France; 4 Institut de Systématique, Evolution, Biodiversité (ISYEB), MNHN, CNRS, Sorbonne Université, EPHE, Université des Antilles, Paris, France Sorbonne Université Paris France; 5 Faculty of Agronomy, Southern Horticultural Research Institute, Tien Giang, Vietnam Faculty of Agronomy, Southern Horticultural Research Institute Tien Giang Vietnam; 6 Vietnam National Museum of Nature, Vietnam Academy of Science and Technology, 18 Hoang Quoc Viet, Cau Giay, Hanoi, Vietnam Vietnam National Museum of Nature, Vietnam Academy of Science and Technology Hanoi Vietnam; 7 Royal Botanical Gardens, Kew, Richmond, UK Royal Botanical Gardens Richmond United Kingdom; 8 University of Agriculture and Forestry, Hue University, Hue, Vietnam Hue University Hue Vietnam; 9 Phu Xuan University, Hue, Vietnam University of Agriculture and Forestry Hue Vietnam; 10 Department of Landscaping and Environmental Horticulture, Nong Lam University, Ho Chi Minh, Vietnam Phu Xuan University Hue Vietnam; 11 Mien Trung Institute for Scientific Research, Vietnam National Museum of Nature, Vietnam Academy of Science and Technology, Hue, Vietnam Nong Lam University, Department of Landscaping and Environmental Horticulture Ho Chi Minh Vietnam; 12 CIRAD, UMR PVBMT, College of Agriculture, Can Tho University, Can Tho, Vietnam Vietnam National Museum of Nature, Vietnam Academy of Science and Technology Hue Vietnam; 13 CIRAD, UMR PHIM, Montpellier, France CIRAD, UMR PHIM Montpellier France; 14 PHIM, University of Montpellier, CIRAD, INRAE, Institut Agro, IRD, Montpellier, France University of Montpellier Montpellier France

**Keywords:** Diversity, *Hiptageaptera*, introduction route, invasive plant, wingless fruit

## Abstract

*Hiptage* (Malpighiaceae) is a genus almost exclusively native to Asia, with a single species, *Hiptagebenghalensis*, being a noxious invasive plant in several areas of the world and particularly threatening the native flora of the Mascarene Islands. Whereas 17 *Hiptage* species have been reported from Vietnam, there is currently no available genetic information that could be used to confirm the species diversity in the country. Through a study combining phylogenetic and morphological analyses of newly-collected samples and herbarium specimens, we were able to accurately identify five *Hiptage* species in Vietnam. One of these, *Hiptageaptera*, represents a species new to science. Specimens of the new species show white to slightly pink petals, erect to forward-curved petals, eight tiny calyx glands and wingless fruits, a unique feature within this genus. Our results strongly support Vietnam as one of the diversity centres of the *Hiptage* genus with 18 species out of the 48 species. Although we could not determine the source of introduction of *Hiptage* in the Mascarenes Archipelago, our findings highlight the genetic similarity of all *Hiptagebenghalensis* samples collected in its secondary distribution areas, suggesting a probable single introduction. This is consistent with historical reports dating the plant’s introduction to the Mascarenes to the 18^th^ century.

## ﻿Introduction

*Hiptage* Gaertn. (Hiptageae, Malpighioideae, Malpighiaceae) is a genus that gathered lianas or shrubs (de Almeida et al. 2024) from, up to now, a total of 47 accepted species (POWO 2024). All species are native to Asian countries, except *H.myrtifolia* A.Gray which is native to Fiji. The most recent common ancestor of *Hiptage* probably arose in the rainforests of Indochina ca. 23 Mya and diversified in this region (de Almeida and van den Berg 2022). The most distinctive feature of the genus is probably the three-winged mericarps whose shape helps seed dispersal through wind and water. The genus is also characterised by many-flowered thyrses, which bear mirror-image flowers (Ren et al. 2013; Qian and Ren 2016). Mirror-image flowers are the result of a kind of sexual polymorphism, known as enantiostyly, where the female reproductive organs are shifted on one side, while the large stamen is shifted to the opposite side. It promotes cross-pollination between flower types and minimises self-pollination (Barrett et al. 2000; Jesson and Barrett 2002; Ren et al. 2013).

The current classification (Arènes 1954) is based on the presence and number of sepal glands that secrete oil as a reward to pollinators, with H.sect.Hiptage (one sepal nectar gland), H.sect.Archihiptage (two to ten sepal nectar glands) and H.sect.Metahiptage (no sepal gland). However, the presence and number of sepal glands are not synapomorphic characters and should, therefore, be used with caution (de Almeida and van den Berg 2022) and morphological analysis should be combined with molecular analysis to improve the reliability of classification.

*Hiptagebenghalensis* (L.) Kurz is the most studied *Hiptage* species native to India and Southeast Asia (POWO 2024). This species rapidly became invasive after its introduction in La Réunion and Mauritius (Friedmann 2011), Florida and Hawaii (Randall 2002; Starr et al. 2003) and northern Australia (Queensland) (Csurhes 2016). Invasions of *H.benghalensis* result in severe negative impacts on the biodiversity of the new areas and the plant is recognised by the IUCN as one of the world’s 100 worst invasive species (Lowe et al. 2000).

Vietnam is recognised as a major centre of the diversity for the *Hiptage* genus (Ren 2015), with 17 species recorded (Pham 2003; POWO 2024). While there are botanical descriptions from French botanists dating back to the mid-19^th^ century (e.g. Pierre 1892) and more recent studies focusing primarily on *H.benghalensis* (Nguyen 2003; Do et al. 2006; Chau et al. 2022), there remains a significant knowledge gap with no genetic information currently available for any specimens sampled in the country. This lack of data not only hinders the characterisation of the rich species diversity in the country, but also limits our knowledge of the genus’s history. Here, in order to obtain a better understanding of the characteristics and distributions of *Hiptage* species in Vietnam, we used ecological niche modelling to predict the potential genus distribution areas and support field surveys and specimen collections. Then, using a combination of morphological and molecular analyses, we determine the diversity of *Hiptage* species in Vietnam and retrace the distribution history of *H.benghalensis*. Our study also revealed a new species of *Hiptage* that is characterised by a wingless fruit, which has not been recorded in all the known *Hiptage* species (Pham 2003; POWO 2024).

## ﻿Materials and methods

### ﻿*Hiptage* niche modelling

To provide information for the field sampling, areas where plants of the genus *Hiptage* are likely to grow were defined using the niche modelling approaches implemented in Biomod2 (Thuiller et al. 2009). Occurrences of *Hiptage* individuals were obtained from GBIF on 27 June 2024 (GBIF.org 2024). It was filtered to keep individuals observed in Indo-Asia, the native area of the genus and those for which full GPS coordinates were available. The filtered dataset comprised 1,613 occurrences from 27 species. Then, using these occurrences along with climatic data from Worldclim 2 (Fick and Hijmans 2017), individual GLM, SRE, RF and MAXNET models were constructed using the SRE pseudo-absence strategy. The final ensemble model was obtained using the weighted mean method and the ROC and TSS metrics. Briefly, it implies that the better an individual model is at predicting the observed distribution, the more importance it has in the ensemble. All other parameters were kept at default or adjusted according to the Biomod2 recommendations available online (https://CRAN.R-project.org/package=biomod2). The model was projected onto Vietnam to identify the most likely locations for *Hiptage*.

### ﻿Sample collection

Silica-dried materials were used in this study from: (i) 44 samples collected during field surveys in Vietnam from 2022 to 2024, (ii) nine samples collected in La Réunion from 2021 to 2023 and (iii) twelve herbarium specimens kept at the Muséum national d’Histoire naturelle de Paris (MNHN) which were collected between 1925 and 2004 from the following countries: China (n = 1), Laos (n = 1), Mauritius (n = 1), Seychelles (n = 3), Sri Lanka (n = 1), Thailand (n = 2) and Vietnam (n = 3). Details on all the samples are provided in Table 1.

**Table 1.** Information on samples collected for this study.

Taxon name	Location	GenBank accession	Year	Geographical coordinates	Original_ID
*Hiptagecandicans*	M’Drak District, Dak Lak Province, Vietnam	PQ449707	2022	12.715697, 108.678551	01_220324_Daklak
*Hiptagelucida*	Ninh Hoa District, Khanh Hoa Province, Vietnam	PQ449709	2022	12.453306, 108.999278	03_220512_KhanhHoa
	Long Dien District, Ba Ria – Vung Tau Province, Vietnam	PQ449710	2022	10.457866, 107.227425	04_220516_VungTau
	Long Dien District, Ba Ria – Vung Tau Province, Vietnam	PQ449711	2022	10.435851, 107.235940	06_220516_VungTau
	Dat Do District, Ba Ria – Vung Tau Province, Vietnam	PQ449712	2022	10.427643, 107.254545	07_220516_VungTau
	Cam Lam District, Khanh Hoa Province, Vietnam	PQ449716	2022	12.183255, 109.118034	14_220707_KhanhHoa
	Cam Lam District, Khanh Hoa Province, Vietnam	PQ449726	2023	12.1320256, 109.0358764	37_230625_KhanhHoa
*Hiptageaptera*	Dat Do District, Ba Ria – Vung Tau Province, Vietnam	PQ449713	2024	10.427317, 107.254949	08_240118_VungTau
	Da Krong District, Quang Tri Province, Vietnam	PQ449718	2022	16.328694, 106.997167	19_221201_QuangTri
	Dat Do District, Ba Ria – Vung Tau Province, Vietnam	PQ449744	2024	10.4064358, 107.2631240	61_240408_VungTau
	Dat Do District, Ba Ria – Vung Tau Province, Vietnam	PQ449745	2024	10.406327, 107.263000	62_240601_VungTau
	Tan Thanh District, Ba Ria – Vung Tau Province, Vietnam	PQ449746	2024	10.545323, 107.147117	64_240623_VungTau
	Dat Do District, Ba Ria – Vung Tau Province, Vietnam	PQ449747	2024	10.433306, 107.254722	65_240623_VungTau
*Hiptagebenghalensis*	Van Yen District, Yen Bai Province, Vietnam	PQ449714	2022	22.005214, 104.535869	11_220528_YenBai
	Van Yen District, Yen Bai Province, Vietnam	PQ449715	2022	22.005436, 104.536119	12_220528_YenBai
	Quang Hoa District, Cao Bang Province, Vietnam	PQ449720	2023	22.756388, 106.296883	29_230414_CaoBang
	Nguyen Binh District, Cao Bang Province, Vietnam	PQ449721	2023	22.691579, 106.005490	30_230414_CaoBang
	Thuan Chau District, Son La Province, Vietnam	PQ449737	2023	21.353379, 103.521779	52_231028_SonLa
	Van Ho District, Son La Province, Vietnam	PQ449738	2023	20.692482, 104.759387	53_231030_SonLa
	Quang Hoa District, Cao Bang Province, Vietnam	PQ449740	2024	22.756117, 106.295807	57_240402_CaoBang
	Quang Hoa District, Cao Bang Province, Vietnam	PQ449741	2024	22.756620, 106.295090	58_240402_CaoBang
	Quang Hoa District, Cao Bang Province, Vietnam	PQ449742	2024	22.757603, 106.294136	59_240402_CaoBang
	Van Yen District, Yen Bai Province, Vietnam	PQ449743	2024	22.005028, 104.535880	60_240404_YenBai
	Hahé Province, Seychelles	PQ449755	1983	-	MNHN_P00904869
	Hahé Province, Seychelles	PQ449756	1983	-	MNHN_P00904870
	Jardin botanique Mahé, Hahé Province, Seychelles	PQ449757	1982	-	MNHN_P01033130
	Laos	PQ449759	1925	-	MNHN_P04783895
	West flank of Piton du Fouge, Mauritius	PQ449760	1975	-	MNHN_P04868852
	Cuc Phuong National Park, Nho Quan District, Ninh Binh Province, Vietnam	PQ449762	2002	20.38472, 105.52583	MNHN_P05474376
	East of Urugala, Kandy District, Central Province, Sri Lanka	PQ449763	1973	-	MNHN_P05598587
	La Grande Chaloupe, La Possession, La Réunion	PQ449724	2023	-20.917837, 55.375325	35_Reunion_2303
	Bassin Boeuf, Sainte-Suzanne, La Réunion	PQ449766	2021	-20.9480831, 55.5836894	REU_BassinBoeuf_1
	Bassin Boeuf, Sainte-Suzanne, La Réunion	PQ449767	2021	-20.9480831, 55.5836894	REU_BassinBoeuf_1bis
	Bassin Boeuf, Sainte-Suzanne, La Réunion	PQ449768	2021	-20.9480831, 55.5836894	REU_BassinBoeuf_2
	La Grande Chaloupe, La Possession, La Réunion	PQ449769	2021	-20.9249947, 55.3793603	REU_GrandeChaloupe_1
	La Grande Chaloupe, La Possession, La Réunion	PQ449770	2021	-20.9249947, 55.3793603	REU_GrandeChaloupe_2
	La Grande Chaloupe, La Possession, La Réunion	PQ449771	2021	-20.9249947, 55.3793603	REU_GrandeChaloupe_3
	Le Petit Serré, Saint-Louis, La Réunion	PQ449772	2021	-21.2230382, 55.4553552	REU_PetitSerre_1
	Le Petit Serré, Saint-Louis, La Réunion	PQ449773	2021	-21.2230382, 55.4553552	REU_PetitSerre_2
*Hiptagemarginata*	Ba Thuoc District, Thanh Hoa Province, Vietnam	PQ449722	2023	20.442347, 105.255294	31_230414_ThanhHoa
	Chu Mon Ray National Park, Sa Thay District, Kon Tum Province, Vietnam	PQ449723	2023	14.48376, 107.71844	33_230414_KonTum
	Cam Lam District, Khanh Hoa Province, Vietnam	PQ449725	2023	12.129393, 109.016691	36_230624_KhanhHoa
	Ninh Hai District, Ninh Thuan Province, Vietnam	PQ449728	2023	11.719153, 109.135425	39_230928_NinhThuan
	Ninh Hai District, Ninh Thuan Province, Vietnam	PQ449730	2023	11.721027, 109.134633	42_230925_NinhThuan
	Ninh Hai District, Ninh Thuan Province, Vietnam	PQ449731	2023	11.721939, 109.136300	43_230929_NinhThuan
	Ninh Hai District, Ninh Thuan Province, Vietnam	PQ449732	2023	11.722832, 109.136383	44_230929_NinhThuan
	Ninh Hai District, Ninh Thuan Province, Vietnam	PQ449733	2023	11.722823, 109.136349	45_230929_NinhThuan
*Hiptagemarginata*	Cam Lam District, Khanh Hoa Province, Vietnam	PQ449739	2023	12.133628, 109.012086	54_231228_KhanhHoa
	Huong Hoa District, Quang Tri Province, Vietnam	PQ449748	2024	16.755854, 106.572304	66_240702_QuangTri
	Huong Hoa District, Quang Tri Province, Vietnam	PQ449749	2024	16.769052, 106.575554	68_240702_QuangTri
	Huong Hoa District, Quang Tri Province, Vietnam	PQ449750	2024	16.769215, 106.575607	69_240702_QuangTri
	Huong Hoa District, Quang Tri Province, Vietnam	PQ449751	2024	16.769437, 106.575694	70_240702_QuangTri
	Chu Mon Ray National Park, Sa Thay District, Kon Tum Province, Vietnam	PQ449752	2024	14.49595, 107.71951	78_240704_KonTum
	Chu Mon Ray National Park, Sa Thay District, Kon Tum Province, Vietnam	PQ449753	2024	14.49409, 107.71908	79_240704_KonTum
	Muong Hoong commune, Dak Glei District, Kon Tum Province, Vietnam	PQ449765	1995	-	MNHN_P05598601
*Hiptage* sp.	Huong Hoa District, Quang Tri Province, Vietnam	PQ449708	2022	16.768469, 106.575449	02_220404_QuangTri
	Ba Thuoc District, Thanh Hoa Province, Vietnam	PQ449717	2022	20.427076, 105.237108	16_220815_ThanhHoa
	My Duc District, Ha Noi Capital, Vietnam	PQ449734	2023	20.614892, 105.765541	49_231102_HaNoi
	My Duc District, Ha Noi Capital, Vietnam	PQ449735	2023	20.614629, 105.765330	50_231102_HaNoi
	My Duc District, Ha Noi Capital, Vietnam	PQ449736	2023	20.614104, 105.764389	51_231102_HaNoi
	Cuc Phuong National Park, Nho Quan District, Ninh Binh Province, Vietnam	PQ449754	2004	20.25417, 105.69528	MNHN_P00819738
	Sam Mo Watt village, Kan-en District, Hainan, China	PQ449758	1934	-	MNHN_P04783883
	Suan Dtoon Falls, Muang District, Songkla Province, Thailand	PQ449761	1985	-	MNHN_P05474375
	Surat District, Kao Wong Province, Thailand	PQ449764	1930	-	MNHN_P05598593
*Chlorohiptagevietnamensis*	Cam Lo District, Quang Tri Province, Vietnam	PQ449727	2023	16.780135, 106.853325	38_2307_QuangTri

### ﻿Morphological analysis

Observations of morphological characters of the species collected in Vietnam were based on either fresh or herbarium specimens depending on their availability. For the new species, each morphological character was annotated during fieldwork and measured in detail. The macro-morphological features were studied, based on the notes in the field and micro-morphological observations were analysed and photographed using an Olympus Stereomicroscope SZX7. The morphological characters of studied specimens were compared with type specimens and protologues of the known *Hiptage* species (at the Herbarium Vietnam National Museum of Nature – VNMN or consulted online on Muséum national d’Histoire naturelle de Paris – MNHN online database, JSTOR Global Plants database; http://plants.jstor.org and the Chinese Virtual Herbarium; http://www.cvh.ac.cn). Taxonomic literature and morphological terminology followed Kurz (1874), Hooker (1875), Arènes (1943, 1954), Jacobs (1955), Sirirugsa (1991), Hajra et al. (1997), Pham (2003), Chen and Funston (2008), Ren et al. (2013), Ren (2015), Yang et al. (2018), Tan et al. (2019), Dong et al. (2020) and Zhang et al. (2023). The conservation status assessment of the new species was based on the International Union for Conservation of Nature guidelines (IUCN 2024).

### ﻿Molecular analysis

For the samples collected during field surveys in Vietnam, total DNA was extracted from dried leaf samples using the CTAB method (Doyle and Doyle 1990). The DNA quantity and quality were measured using an Eppendorf BioPhotometer D.30 spectrophotometer (Eppendorf, Germany). Samples with DNA concentrations above 10 ng/µl and A_260 nm_/A_280 nm_ ratio in the range of 1.8 to 2.0 were used for further analyses. DNA extracts were stored at -20 °C before use. Amplicons of the nuclear ribosomal Internal Transcribed Spacer (ITS) were then obtained using the primers ITS5A (Stanford et al. 2000) and ITS4 (White et al. 1990) using either MyTaq DNA Polymerase (Bioline, Meridian Life Science) or 2X Taq Master Mix (dye plus) (Vazyme Biotech Co.). The PCR reactions were established following the manufacturer’s recommendations. PCR cycles were initial denaturation for 5 min at 95 °C, followed with 40 cycles with 95 °C for 30 sec, 58 °C for 30 sec and 72 °C for 30 sec before a final elongation for 5 min at 72 °C. In order to confirm the presence of amplicons of the expected size (~ 650 bp), PCR products were visualised using UV light after agarose gel electrophoresis and ethidium bromide staining. PCR amplicons were sent to Macrogen Company (South Korea) and Phusa Company (Vietnam) for direct sequencing in both directions. Chromatograms were then assembled using DNA Baser Sequence Assembler v.5.21.0 (Heracle BioSoft, Romania). All ambiguous positions were converted into IUPAC degenerate nucleotide codes.

For the herbarium samples, total plant DNA was extracted from 20 mg of dried leaves with DNeasy Plant Mini Kits (Qiagen). The DNA isolates were assessed by electrophoresis and their concentrations were measured by Qubit^TM^ dsDNA High Sensitivity Kits (Invitrogen). All twelve herbarium DNAs were purified with the Nucleomag NGS Clean-up and Size Select Kit (Macherey-Nagel). ITS amplification was performed using the primers ITS1P (Ridgway et al. 2003) and ITS4 (White et al. 1990) and LongAmp® Taq DNA Polymerase (New England Biolabs). Samples underwent PCR as follows: denaturation for 30 sec at 94 °C, 35 cycles with 30 sec at 94 °C, 55 sec at 50 °C, 1 min at 65 °C, then 15 minutes at 65 °C. PCR amplification was controlled by electrophoresis, then PCR amplicons were sent to Eurofins (France) for Sanger sequencing in both directions.

### ﻿Phylogenetic analysis

A total of 52 ITS sequences from 21 *Hiptage* species and five ITS sequences of *Chlorohiptage*, *Callaeum*, *Heteropterys* and *Niedenzuella* genera, used as outgroups, were obtained from NCBI in January 2024 (Suppl. material 1). These 57 sequences, along with the 65 new sequences obtained in this study, were aligned using MAFFT v.7.520 (Katoh and Standley 2013). A phylogenetic tree was inferred with 1,000 bootstrap replicates using IQ-TREE v.2.2.6 (Minh et al. 2020). Using the automatic model selection procedure implemented in IQ-TREE, the TNe+R2 model was selected as the best-fitted following the Bayesian Information Criterion. The ML tree was visualised and annotated using the ‘plot.phylo’ function from the ‘ape’ R package (Paradis and Schliep 2019).

## ﻿Results

### ﻿Assessment of the *Hiptage* distributions in Vietnam

**Figure 1.**
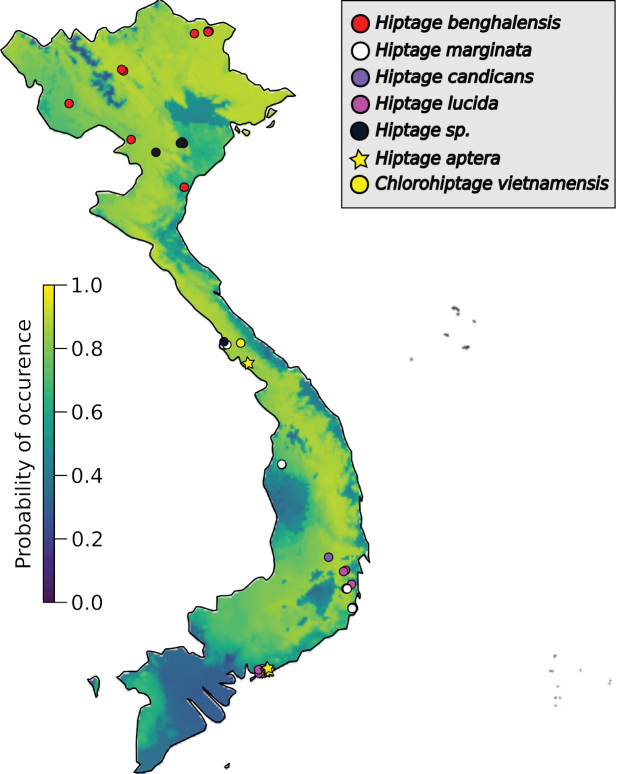
Vietnam map showing locations of the *Hiptage* samples collected during this study. Samples are coloured according to the species, as described on the legend on top-right. Probabilities of the occurrence of *Hiptage* in Vietnam, inferred using Biomod2 (Thuiller et al. 2023) and WorldClim 2 bioclimatic variables (Fick and Hijmans 2017), are represented on the background of the map and are coloured according to the legend on the left of the map. The yellowish areas indicate climatic conditions highly favourable for *Hiptage*.

Whereas the *Hiptage* genus has a large geographical distribution, proper knowledge of the distribution of that genus in Vietnam was lacking. In order to assess the areas of Vietnam, a country that spans more than 14° of latitude, which may be most suitable for plants of *Hiptage*, we conducted niche modelling analyses. The analyses demonstrated that most of the Vietnamese regions (except for the Mekong Delta and some local areas) have very favourable climatic conditions for *Hiptage* (see the yellowish colour on the background of Fig. 1), so that a pan-country distribution of the genus could be expected. Accordingly, *Hiptage* samples were collected in forest ecosystems from montane and coastal areas or growing as wild or ornamental plants in urban areas from eleven provinces across Vietnam.

As most *Hiptage* species only flower seasonally (Pham 2003; Chen and Funston 2008), typically in March–April each year, we could not gather all the necessary morphological data to identify the species. Indeed, out of the 44 samples collected in Vietnam during the survey, complete morphological characteristics were obtained for 14 samples. Our identification would, thus, rely on molecular analysis. In total, including samples collected during this study in Vietnam, La Réunion and the herbarium specimens, we obtained 65 ITS sequences. All the details of these samples and the accession numbers of the ITS sequences are available in Table 1.

### ﻿Sequences obtained from the herbaria samples

**Figure 2.**
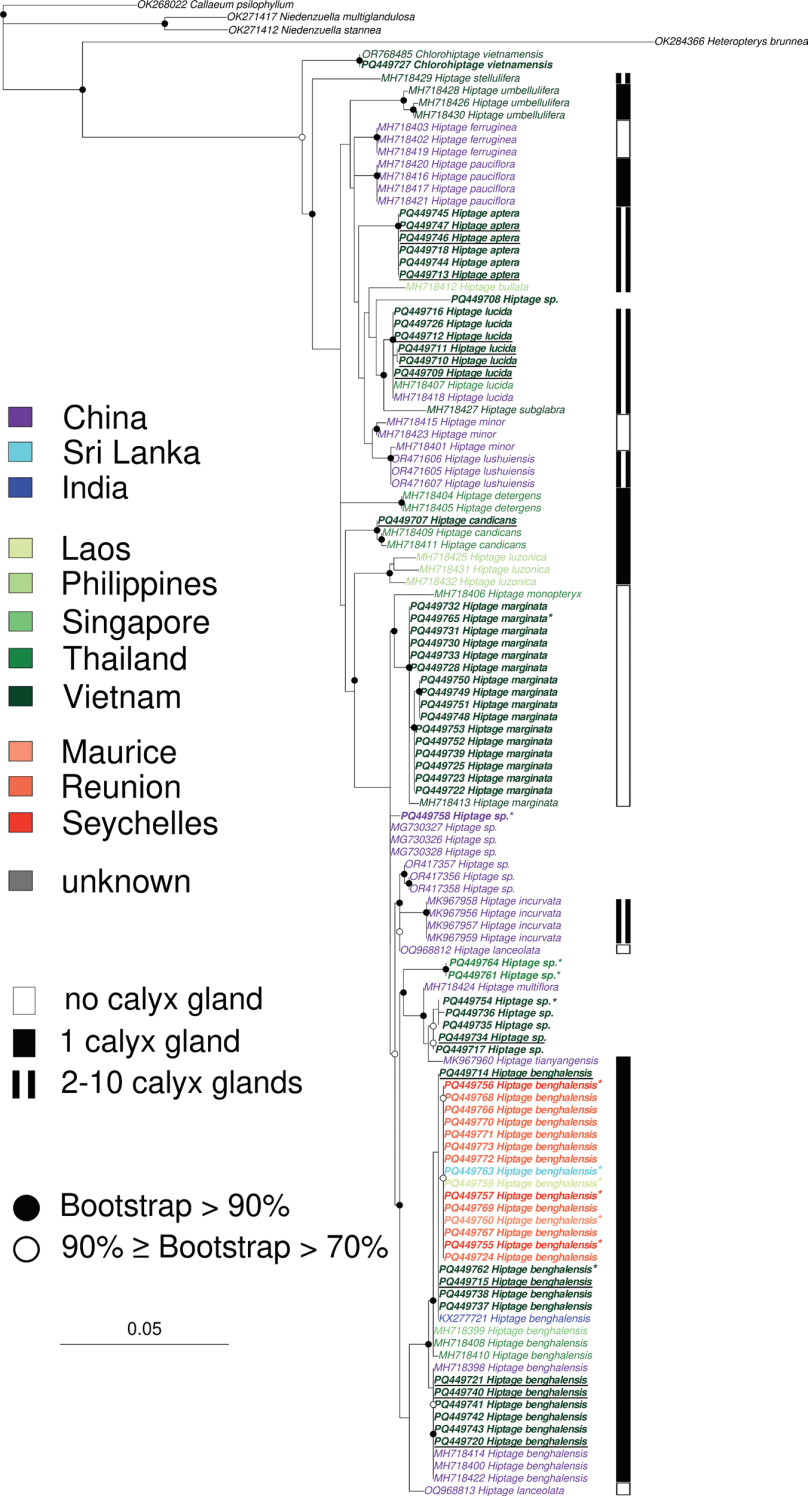
Phylogenetic relationships of *Hiptage*, based on ITS sequences. Colours represent the origins of the sequences according to the legend shown at the top left of the tree. The presence and number of the calyx glands is indicated on the right of the tree tips according to the legend shown at the bottom left of the tree. Sequences obtained in this study are in bold and sequences from herbaria are indicated with a star (*). Sequences from this study with full morphological characteristics are underlined. Open and closed circles on nodes indicate bootstrap support for the branches to their left, superior to 70% and to 90%, respectively.

Twelve sequences were obtained from herbaria samples labelled as *H.benghalensis* and deposited at MNHN (Table 1). These samples were collected from 1925 to 2004 from seven countries. Congruent with their labelling in the collection, seven of the samples were clearly clustering within the *H.benghalensis* clade with sequences from Mauritius, Laos, Seychelles, Sri Lanka and Vietnam. All these sequences were collected between 1925 and 2002. Interestingly, the other five sequences were grouping with other *Hiptage* species (Fig. 2). Amongst these, one sequence (PQ449765) from a sample collected in Vietnam in 1995 was clustering with *H.marginata*. Conversely, the four other sequences appeared as outgroups of currently identified species: one sequence (PQ449754) collected in Vietnam in 2004 was very close to the sequences of *H.tianyangensis* (MK967960) which is known endemically in China. Two sequences (PQ449764; PQ449761), obtained from Thailand and collected in 1930 and 1985, were forming a sister clade of *H.multiflora* and *H.tianyangensis*. The last sequence (PQ449758), obtained from a sample collected in China in 1934, was more closely related to three other sequences (MG730326–MG730328) also obtained from China and identified in GenBank as *H.benghalensis*. However, as these sequences clearly fall outside the *H.benghalensis* clade, they would most probably belong to another species.

### ﻿Sequences of *H.benghalensis* in the world

Of all the 26 *H.benghalensis* sequences analysed in this study, four sequence groups were identified. Whereas all the groups gathered sequences from Southeast Asia, all the sequences that were obtained from the Mascarenes and Seychelles archipelagos (reddish-coloured tips on Fig. 2), one of the main invasive areas, were grouped into a single clade. This clade gathered other sequences from samples collected in Laos and Sri Lanka, both from herbaria. These sequences were indeed 100% identical to one another. The *H.benghalensis* sequences from Vietnam were found in three groups: one that included additional samples from China, another that encompassed sequences from Thailand and Singapore and the third group consisting of sequences from Vietnam (including one from herbaria) alongside a sequence from India; none was associated with samples from the invasive area.

### ﻿*Hiptageaptera* sp. nov. – a new species with wingless fruits

Sequences obtained from six samples collected in three localities in Vietnam exhibited clear genetic differences from the known *Hiptage* species (Fig. 2). These sequences form a distinct clade, supported by 100% of bootstrap replicates. Amongst the species and specimens analysed, *H.minor* shows the closest phylogenetic distance to the proposed new species and is also closely related to *H.lushuiensis*, *H.bullata*, *H.subglabra*, *H.lucida* and other unknown species. This new species exhibits diverse leaf shapes, similar to those of the comparison species. All possess basal leaf glands and several small calyx glands. However, they differ in the number of calyx glands and *H.minor* lacks calyx glands entirely. In addition, the sample of this new species were clearly distinguished from the related species by their unique wingless fruits and were, thus, proposed as belonging to a new species, namely *Hiptageaptera* M.T.X.Lam & T.V.Do, sp. nov.. The distinguishing features are presented in Table 2.

**Table 2.** Morphological comparison of key characteristics in *H.aptera*, *H.lushuiensis*, *H.minor*, *H.bullata*, *H.subglabra*, *H.lucida* and *H.benghalensis*.

Character	*Hiptageaptera*	*Hiptagelushuiensis* ^1^	*Hiptageminor* ^2^	*Hiptagebullata* ^3^	*Hiptagesubglabra* ^4^	*Hiptagelucida* ^5^	*Hiptagebenghalensis* ^6,7,8,9^
Leaf blade	oblong, elliptic, ovate to lanceolate, 5.5–17.5 cm long	elliptic, 9–16 cm long	ovate-lanceolate, small, 1.2–7.6 cm long	ovate, ovate-elliptic to ovate-oblong, 4.5–14.5 cm long	ovate-oblong or ovate-elliptic, lanceolate, leathery, 3–10 cm long	ovate-oblong or ovate-elliptic; leathery, thick, 9–10 cm long	oblong, eliptic-oblong, or ovate-lanceolate, 7.6–21 cm long
Branchelet	red brown pubescent	white to grey sericeous	puberulent	-	-	pubescent	densely yellowish brown or silver-gray pubescent
Leaf base	lanceolate, oblong, orbicular, ovate to rotound, cuneate	cuneate	cuneate	rounded to sub-cordate	rounded	rounded	acute to rounded, cuneate, broadly cuneate or obtuse
Leaf apex	accumninate or retuse	acuminate	acuminate	mucronate or acuminate, rarely obtuse	rather abruptly attenuate-obtuse	obtuse rigid tip	acuminate, rarely acute or rounded
Leaf basal glands	1–3 pairs	1 pair	1 pair	often 1 pair	1 pair	1 pair	often 1 pair
Thyrses	axillary or terminal; 2.5–20 cm long	axillary or terminal, 3–10 cm long	axillary and terminal	4–13 cm long	axillary, 5–10 cm long	axillary and terminal, 12 cm long	axillary or terminal, 4–35 cm long
Sepals	ovate, apex rounded to acute, ca. 15 mm long	elliptic, apex obtuse, 4–5 mm long	orbicular, apex obtuse, ca. 2 mm long	ovate or oblong, apex rounded, ca. 3 mm long	ovate, apex rounded, ca. 2.5 mm long	apex rounded	obtuse, broadly elliptic, ovate or oblong, 2–10 mm long
Calyx glands	8 glands, small, ca. 0.5 mm long	2 glands, sometimes with additional smaller glands, ca 1 mm long	absent	usually 2 glands on each sepal, less than 1 mm long	2–5 glands	1–2 small glands between each sepal	only 1 gland, very large
Petals	white to light pink, suborbicular, erect to forward curved, 10–14 × 8–12 mm, fimbriated	pink, suborbicular, extremely reflexed, ca. 10 mm long, clawed	white, rounded plates, erect to forward curved, fimbriated	white, suborbicular, 8 × 6 mm, clawed	white, suborbicular, erect to forward curved, 15 mm long, clawed	white or pink, erect to forward curved, 10 mm long, unfimbriated	white, ovate-oblong to suborbicular, extremely reflexed, 8–15 mm long, fimbriated, clawed
Fruits	wingless, hemispherical	3-winged	3-winged	3-winged	-	3-winged	3-winged

(1) Dong et al. (2020); (2) Chen and Funston (2008); (3) Sirirugsa (1991); (4) Arènes (1943); (5) Pierre and Doin (1880); (6) Kurz (1874); (7) Hajra et al. (1997); (8) Chen and Funston (2008); (9) Pham (2003).

#### ﻿ Hiptage aptera

Taxon classificationPlantaeMalpighialesMalpighiaceae

M.T.X.Lam & T.V.Do sp. nov.

220B8B1A-C370-58DD-8FAB-C601BE32710C

urn:lsid:ipni.org:names:77362501-1

##### Type.

Vietnam: • Ba Ria–Vung Tau Province, Dat Do District, 10.427317, 107.254949, 39 m elev., 16 May 2022, *Nguyen Hoang Nam 08_240118_VungTau* (holotype: VNMN; isotype: VNMN).

##### Diagnosis.

This species shares similarities with *H.lushuiensis*, *H.minor*, *H.bullata*, *H.subglabra* and *H.lucida* such as leaf shape, basal leaf glands and petal colour. However, *H.aptera* differs from these related species in petal size and calyx gland presence. Its petals are significantly larger than those of *H.lushuiensis*, *H.minor*, *H.bullata* and *H.subglabra* and are erect to forward-curved with fimbriate margins, unlike the strongly reflexed petals in *H.lushuiensis* and the unfimbriate petals in *H.lucida.* Additionally, the absence of calyx glands distinguishes *H.aptera* from *H.minor*. This new species is distinctly different from *H.benghalensis*, one of the most studied species in the genus, which has extremely reflexed petals and a large calyx gland. A notable feature of *H.aptera* is its wingless, hemispherical mericarps (fruits), which are relatively large, measuring 2.4–4.5 cm in diameter. This unique fruit shape represents the first recorded occurrence within the *Hiptage* genus.

##### Description.

**Figure 3.**
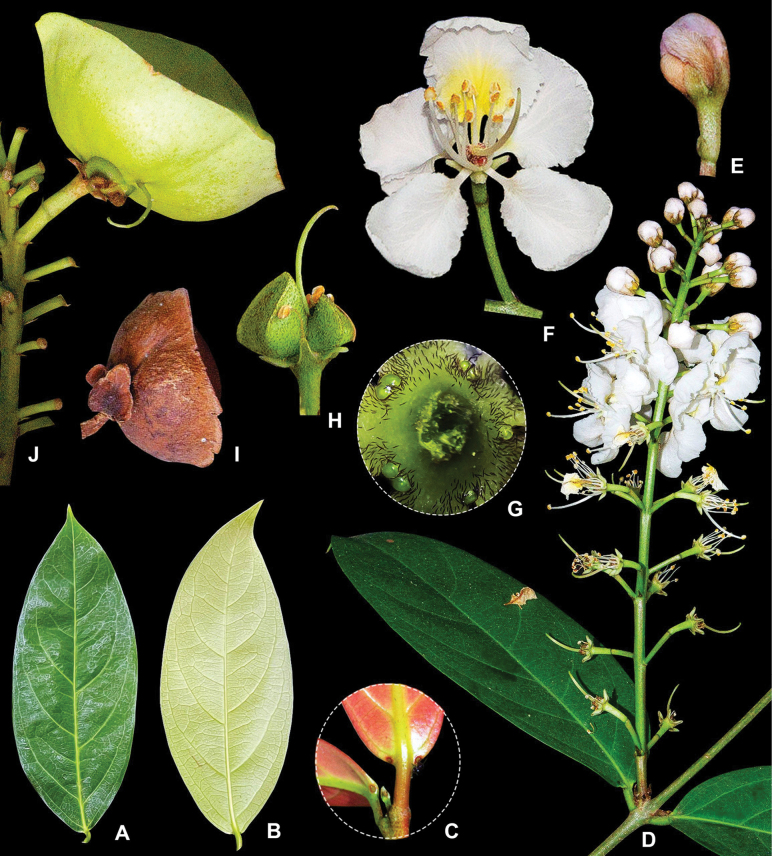
Morphological characteristics of *Hiptageaptera* M.T.X.Lam & T.V.Do, sp. nov. with wingless fruits **A, B** adaxial and abaxial surface of leaf **C** two basal glands of leaf **D** flowering branch **E** bud of flower **F** flower **G** calyx glands **H–J** wingless fruits. Photos by Lam Thi Xuan Mai and Nguyen Hoang Nam, edited by Rafael Felipe de Almeida.

Woody shrubs or lianas, young branches densely red-brown pubescent, hairs appressed; older twigs glabrous, with white and small lenticels, rounded, coarse, stem wart-like structures. ***Leaves*** opposite; stipules present, linear, 1–2 mm, located at the base of the petiole, densely red-brown pubescent; petioles ca. 6 mm long, densely red-brown pubescent, adaxially canaliculate; leaf blade oblong, elliptic, ovate to lanceolate, 5.5–17.5 × 2–7 cm, coriaceous; young leaves usually red, white or red-brown pubescent on abaxial surface, densely so along mid-rib, adaxially glabrous; mature leaves green, glabrous on both surfaces, base cuneate, apex acuminate or retuse, margin entire, abaxially often with 1–3 pairs marginal gland near the base; lateral veins 5–7 pairs, prominent on both surfaces. ***Thyrses***: terminal or axillary; main axis 2.5–20 cm long, red-brown pubescent; peduncle 2–17 mm long, covered red-brown pubescent; bracts lanceolate, 9–12 mm long, green; bracteoles lanceolate, 2–3 mm long, red-brown. ***Flowers***: white to slightly pink; pedicels 16–22 mm long, spread red-brown pubescent. ***Calyx*** with five sepals basally connate, ovate to round, apex rounded to acute, ca. 3 × 15 mm, densely light brown pubescent abaxially, glabrous adaxially. ***Calyx glands***: eight prominent, round, small, green, not decurrent to the pedicel, often connate at the junction between two sepals. ***Petals***: five, suborbicular, 11–14 × 8–12 mm, white to light pink, erect to forward curved, apex rounded, margin fimbriate, claw short, ca. 0.2 mm length, ca. 0.3 mm width, basally yellow, yellow maculations, abaxially red brown pubescent, densely so near base, abaxially glabrous. ***Stamens*** ten, glabrous, unequal in size; filaments white, circinate, 9–11 mm long in the longest stamen and 2–5 mm long in the remaining nine; anthers oblong; pollen sacs yellow, rimose (longitudinal dehiscence). ***Ovary***: ca. 25 mm, ovoid, densely light red pubescent; style 1, yellowish-green, 7.5–12 mm long, slightly curved upwards, deflected either to the left or right side, glabrous; stigma apical. ***Fruits***: wingless, 1–3 mericarps, hemispherical, spread red brown pubescent, young fruit green, red-brown pubescent on both surfaces; mature fruit rusty to reddish-brown, glabrous, 2.4–4.5 cm in diam., adaxially with longitudinal groove, abaxially bulging. ***Seeds***: ovoid, ca. 1.7 cm long, dark yellow or brown (Fig. 3).

##### Phenology.

Flowering in March to June and fruiting from May to August.

##### Etymology.

The specific epithet reflects the unusual fruit morphology compared to other *Hiptage* species.

##### Vernacular name.

Vietnamese: Dùi đục không cánh. “Dùi đục” is the popular Vietnamese name of *Hiptage* genus, “không cánh” is wingless.

##### English name.

Wingless Hiptage.

##### Habitat and distribution.

*Hiptageaptera* is found in two Provinces: Ba Ria–Vung Tau (southern Vietnam) and Quang Tri (central Vietnam). It grows in areas around the base of mountains, on slopes and along mountain paths under evergreen forests and near residential areas, at elevations of 28–433 m.

##### Conservation status

**(and study).** Since its identification, we have found this species in various locations within Ba Ria–Vung Tau Province, where it maintains a relatively large population. It is commonly observed as shrubs or lianas covering certain areas, co-existing with other species in the same habitat. We propose that the new species *H.aptera* should be classified as Least Concern (LC) according to the current IUCN Red List Categories and Criteria (IUCN 2024). However, continuous monitoring of its population status and habitat conditions is crucial for making accurate assessments in the future.

##### Additional specimens examined

**(*paratypes*).** Vietnam: • Dat Do, Ba Ria–Vung Tau, 10.4064358, 107.2631240, 194 m elev., 8 Apr 2024, Lam Thi Xuan Mai, original ID 61_240408_VungTau; • Dat Do, Ba Ria–Vung Tau, 10.406327, 107.263000, 190 m elev., 01 Jun 2024, Lam Thi Xuan Mai, original ID 62_240601_VungTau; • Tan Thanh, Ba Ria–Vung Tau, 10.545323, 107.147117, 333 m elev., 23 Jun 2024, Nguyen Hoang Nam, original ID 64_240623_VungTau; • Dat Do, Ba Ria–Vung Tau, 10.433306, 107.254722, 28 m elev., 23 Jun 2024, Nguyen Hoang Nam, original ID 65_240623_Vung Tau; • Da Krong, Quang Tri, 16.328694, 106.997167, 433 m elev., 01 Dec 2022, Tran Dang Khoa, original ID 19_221201_QuangTri.

## ﻿Discussion

### ﻿Presence, diversity and distribution of *Hiptage* genus in Vietnam

This study represents the first attempt at molecular characterisation of *Hiptage* species in Vietnam. From our results, four species that have already been known elsewhere and a new species were identified, these *Hiptage* populations being distributed across various localities in Vietnam. Besides, our study also included six unidentified samples. Based on the molecular analysis and despite the absence of morphological description, these samples (sequences PQ449708; PQ449717; PQ449734–PQ449736 and PQ449754; *Hiptage* spp.) were most likely belonging to two unidentified species. It is important to note that Vietnam is home to 17 *Hiptage* species, twelve of which are endemic to the country (POWO 2024). This study updated the count to 18 species, with 13 species currently known exclusively from Vietnam. Whereas our analyses revealed that most of Vietnam presents climatic conditions favourable to plants from the *Hiptage* genus in general, it would now be important from a botanical and conservation perspective to gather more knowledge on these 18 species and their distinct distribution in the country. Resampling during the flowering and fruiting season will be essential for further characterisation. Confronting the morphological reports of these species would reveal how the newly-uncovered clades match or complement the known diversity of *Hiptage* in Vietnam. The presence of known species, a new species and unidentified specimens further supports the notion that the Indochina Peninsula is a hotspot for the genus and Vietnam is a centre of its diversity (Ren 2015; de Almeida and van den Berg 2022).

### ﻿Potential migration route to the South West Indian Ocean Islands

Based on the phylogenetic relatedness of the 13 *H.benghalensis* samples collected in the secondary area, it is interesting to notice first that all the samples fall in the same clade, suggestive of a single introduction event of the plant into the region or all the introductions in all the islands were from the same area. Indeed, based on historical reports (Céré 1785; M.-S. Jagou and F. Martos, pers. comm.), it is thought that the liana was introduced in Mauritius in the late 18^th^ century and later spread to the surrounding islands. Nevertheless, although our analysis did support that hypothesis, it remains difficult to retrace the introduction route to any of the native areas. One sample from Laos and another from Sri Lanka are, indeed, genetically similar to the introduced ones. However, no clear-cut conclusion could be drawn, as determining the origin of any invasive populations often requires more abundant and more intensive sampling from their native range (Hagenblad et al. 2015) and more diverse genetic markers would certainly be required. In addition, even though all the samples collected in Vietnam appeared in other phylogenetic groups, it does not absolutely rule out the possibility that the introduced populations were from that area. It is very likely that other populations with different genetic profiles are present in Vietnam, but were not sampled here. It is also entirely possible that some populations existed in Vietnam at some point, but are now extinct.

Collecting additional samples in Vietnam and from other Southeast Asian countries and the Indian Peninsula would help clarify the migration routes of *H.benghalensis* to the South West Indian Ocean Islands. Our study also points to the requirement for a more discriminant genetic method that would help refine our understanding of sample relationships. Such an initiative would provide invaluable clues on how *H.benghalensis* may have disseminated to the world, where it has become invasive and caused ecological consequences in the introduced areas. It is noteworthy that, in the process of achieving this feat, populations of previously unknown *Hiptage* species were discovered, putting this genus at both extremes of the conservation spectrum.

### ﻿Wingless fruits of *Hiptageaptera* – A morphological innovation in *Hiptage*

The newly-identified species from Vietnam, named *Hiptageaptera*, derives its name from the Greek term “aptera,” which refers to the absence of wings on the fruit. This species presents eight calyx glands, a clear distinctive character from the closest related species in the ITS phylogeny, *H.minor* with no calyx gland or other closely-related species, *H.lushuiensis*, *H.bullata*, *H.subglabra* and *H.lucida*, which differ in the number of calyx glands present. It must be noted, however, that this character is homoplastic in the *Hiptage* genus and can be problematic for taxonomic classification (de Almeida and Van den Berg 2022). Nevertheless, the phylogenetic tree provides strong support for distinguishing this species. The species produces large, hemispherical, wingless fruits known as smooth mericarps. Whereas it represents a first for the *Hiptage* genus, the observation of a mixed winged- and wingless-fruit was also observed in other genera within the family, such as *Camarea*, *Mascagnia* and several other Neotropical genera (Davis and Anderson 2010). Understanding the factors associated with this change would be important. The realistic observation that the fruit was floating on the water points to a possible dispersal through water (hydrochory), which is a very common strategy in Amazonian Malpighiaceae growing near large bodies of water (de Almeida et al. 2024). The transition from winged to wingless fruits with hard pericarps may also be related to a “defence scenario” proposed by Mack (2000). This change enhances the protective ability to defend seeds and secondarily evolves into structures that promote seed dispersal.

## ﻿Conclusion

Our study highlights Vietnam as a significant centre of diversity for the *Hiptage* genus, with 18 species to date, including both previously-recorded species and the newly-described *Hiptageaptera*. The new species is distinct with its unique morphological trait – wingless fruit, contrary to all known *Hiptage* species. Furthermore, while the origin of *H.benghalensis* in the Mascarene Islands remains unclear, our genetic analysis indicated a probable single introduction event, aligning with historical records from the 18^th^ century.

## Supplementary Material

XML Treatment for Hiptage aptera

## ﻿Additional information

### Author ORCIDs

Mai Thi Xuan Lam https://orcid.org/0009-0007-5973-469X

Marie-Stella Jagou https://orcid.org/0009-0009-0351-940X

Florent Martos https://orcid.org/0000-0003-1213-5466

Truong Van Do https://orcid.org/0000-0002-0585-5513

Rafael Felipe de Almeida https://orcid.org/0000-0002-9562-9287

Hoa Dang Tran https://orcid.org/0000-0002-3421-9444

Khoa Dang Tran https://orcid.org/0000-0002-0842-5279

Ky Huynh https://orcid.org/0000-0002-7615-2866

Diep Quang Dinh https://orcid.org/0000-0002-1462-0070

Xuan Thi Trinh https://orcid.org/0009-0002-0513-7922

Jean-Philippe Deguine https://orcid.org/0000-0002-3689-3707

### Data availability

All of the data that support the findings of this study are available in the main text or Supplementary Information.

## Supplementary materials

Supplementary material 1 Taxa and GenBank accession numbers of *Hiptage* used in this study Data type xlsx https://binary.pensoft.net/file/1343371This dataset is made available under the Open Database License (http://opendatacommons.org/licenses/odbl/1.0/). The Open Database License (ODbL) is a license agreement intended to allow users to freely share, modify, and use this Dataset while maintaining this same freedom for others, provided that the original source and author(s) are credited.Mai Thi Xuan Lam, Marie-Stella Jagou, Giang Quoc Van, Florent Martos, Truc Thi Ngoc Nguyen, Truong Van Do, Rafael Felipe de Almeida, Hoa Dang Tran, Khoa Dang Tran, Giang Thi Nguyen, Dong Phuong Tran, Ky Huynh, Jean-Noël Rivière, Mathieu Rouget, Diep Quang Dinh, Anh Tuan Le, Xuan Thi Trinh, Jean-Philippe Deguine, Pierre Lefeuvre
